# Personality Functioning Is Not a Unitary Entity

**DOI:** 10.1002/pmh.70033

**Published:** 2025-06-29

**Authors:** Orestis Zavlis

**Affiliations:** ^1^ University College London London UK

I recently had the pleasure of reading the interesting paper by Ohse et al. (2025). I appreciated the authors' analysis of the disparities and similarities between the contemporary construct of “personality functioning” and its traditional counterpart “personality organization.” At the same time, however, I also wondered whether there is merit in critically assessing an implicit assumption of their work: namely, the view of personality functioning as a unitary entity.

To elaborate, Ohse et al. (2025) predicated their factor analyses on a bifactor technique, thereby assuming that personality functioning can be conceived as a general factor, uncorrelated with a set of specific factors. While this assumption is widely held in the field of personality psychopathology and aligns with the original conceptualization of “personality functioning,” I believe it is important to scrutinize rather than reify this way of viewing personality functioning by testing whether this construct is truly unitary.

To explore this hypothesis, I conducted three analyses, the code for which can be found online. First, I conducted a bootstrapped exploratory graph analysis–––a recently developed factor‐analytic methodology that can identify latent variables from a set of observed variables without the need for factor‐rotation or researcher‐interpretation as traditional factor‐analytic methods suppose (Christensen and Golino 2021; Golino et al. 2020; Golino and Epskamp 2017). Applying this method to the 12 personality functioning subscales and in 1000 bootstrapped samples from the authors' publicly available data (*N* = 277), I found evidence that personality functioning can be bifurcated into two domains: a ***self‐focused domain*** that comprises three identity subscales, three self‐direction subscales, and, notably, one intimacy subscale (reflecting the “desire and capacity for closeness”); and an ***other‐focused domain*** that comprises three empathy subscales and the remaining two intimacy subscales.

Second, confirmatory factor analyses illustrated that this two‐factorial model (which had the same fit with a structure loading all intimacy scales onto the other‐focused domain) provided a significantly better fit to the data (CFI = 0.98, TLI = 0.97, RMSEA = 0.053, SRMR = 0.032) compared to a unidimensional factor model (CFI = 0.88, TLI = 0.85, RMSEA = 0.12, SRMR = 0.062; *χ*2 (7) = 205, *p* < 0.001) which assumes that personality functioning is a unitary entity.

Finally, regression analyses further supported the utility of separating the two personality functioning domains by illustrating that the self‐functioning domain was primarily associated with ***emotional psychopathology*** (showing significant, at *p* < 0.001, associations with identity diffusion (*β* = 0.38), emotional lability (*β* = 0.26), and depressivity (*β* = 0.23)); while the other‐functioning domain was primarily associated with ***social psychopathology*** (showing significant, at *p* < 0.001, associations with maladaptive representations of others (*β* = 0.23), suspiciousness (*β* = 0.16), and withdrawal (*β* = 0.23).

I believe these findings are noteworthy for at least two reasons. First and foremost, they highlight that there is value in moving beyond bifactor models, as these models tend to overfit datasets and impede the development of psychological theories [by focusing on abstract psychological entities (i.e., general factors of personality or psychopathology) that lack substantive meaning (see Fried 2020; Watts et al. 2023; Zavlis and Fried 2024)]. Second, they suggest that there is value in conceptualizing personality functioning in terms of two distinct domains that are clearly interpretable and have a long‐standing foundation in personality theories: namely, the domains of ***autonomy*** (i.e., the capacity to establish an identity and direction in life) and ***relatedness*** (i.e., the capacity to form and maintain meaningful relationships; see Blatt 2008; Hopwood 2018; Luyten and Blatt 2011; Wright et al. 2023; Zavlis 2024). Future research should further scrutinize these domains, particularly in longitudinal and therapeutic settings, to further examine the hypothesis that personality functioning is better understood both theoretically and psychometrically in terms of two distinct domains: *self‐functioning* and *social‐functioning* (e.g., Zavlis 2024; Zavlis, Bentall, et al. 2024; Zavlis, Fonagy, et al. 2024).

## Conflicts of Interest

The author declares no conflicts of interest.

## Data Availablity Statement

Data and Code are openly available here: https://osf.io/a7vwr/.
